# Inter-rater Reliability and Construct Validity of the Lerner Defense Scale in Clinical and Non-clinical Groups

**DOI:** 10.3389/fpsyg.2021.708886

**Published:** 2021-09-24

**Authors:** Anna Maria Rosso, Cinzia Airaldi, Andrea Camoirano

**Affiliations:** ^1^Department of Educational Sciences, University of Genoa, Genoa, Italy; ^2^Associazione Psicologia Clinica, Genova, Italy

**Keywords:** defense mechanisms, Lerner Defense Scale, Rorschach test, Psychodynamic Diagnostic Manual-second edition, level of personality organization

## Abstract

The current study investigated the inter-rater reliability and the construct validity of the Rorschach Lerner Defense Scale (LDS). In particular, it aimed to explore the inter-rater reliability, analyzing the most frequent coding mistakes in an attempt to improve the coding guidelines, and to investigate the ability of the scale to distinguish between individuals with neurotic-level and borderline-level personality organization, according to the Psychodynamic Diagnostic Manual-2 (PDM-2), and non-clinical subjects. Eighty clinical subjects and 80 non-clinical ones participated in the study. Among the clinical subjects, 40 have borderline-level personality organization and 40 have neurotic-level personality organization. Non-clinical subjects were drawn from an archival dataset of non-clinical individuals who previously participated in a Rorschach normative study. The LDS showed substantial inter-rater reliability; however, guidelines could be improved, specifically with regard to the threshold for coding Devaluation and Idealization at level 1. Furthermore, more examples should be included in the manual about the coding of Projective Identification and Denial. The LDS distinguished borderline-level subjects from both the non-clinical and neurotic groups with regard to Devaluation and Projective Identification, with borderline-level personality organization subjects reporting higher scores than either of the two other groups. Only the Denial scale discriminated between the non-clinical and neurotic group, with the latter reporting higher scores of high-level Denial.

## Introduction

In psychoanalysis, the conceptualization of the defenses followed a long path which can be described, briefly and in a reductive way, as the transition from being considered pathogenic elements to becoming protective factors against negative affects (Freud, 1926, 1938). Klein (1946) added that defenses not only protect individuals from painful feelings but also significantly contribute to organizing psychic development.

More recently, Kernberg (1975) identified the quality of the defense mechanisms as a crucial diagnostic criterion for differentiating among neurotic, borderline, and psychotic levels of personality organization. The massive use of primitive splitting and denial was peculiar to the psychotic level, especially if associated with impaired reality testing, while projective identification, primitive idealization, and devaluation were distinctive defense mechanisms used by individuals with a borderline level of personality organization.

Currently, according to the Psychodynamic Diagnostic Manual-2 (PDM-2; Lingiardi and McWilliams, 2017), developed on the basis of Kernberg's theory, defensive functioning is one of the 12 psychological capacities included in the Mental Functioning Axis (M Axis), and its assessment is essential to identify the level of personality organization (P Axis).

Since defenses are unconscious psychic processes, measuring them may be challenging and performance-based tests may be more appropriate for identifying them. As Lerner (2005) stated, the Rorschach can provide psychoanalysts and researchers a way to operationalize psychic processes that are not directly observable. In 1980, Lerner and Lerner developed the Lerner Defense Scale (LDS) based on Kernberg's theoretical framework in order to evaluate the emergence of the primitive defenses of Splitting, Devaluation, Idealization, Projective Identification, and Denial in the Rorschach test.

The theoretical orientation of Lerner and Lerner is at the basis of the choice to consider only Rorschach responses with human content as the unit of analysis. According to Kernberg's theoretical formulation, in fact, there is a close link between defenses and object relations, and in the Rorschach test, the latter is represented in a peculiar way by responses with human content.

The LDS, which is described in detail in the Materials and methods section, showed good levels of reliability between coders, exhibiting agreement rates between 83 and 100% in one study (Lerner and Lerner, 1980) and correlation coefficients between 0.94 and 0.96 in a second study (Lerner et al., 1981). Perry and Ianni (1998) in a more recent review reported high inter-rater reliability with *r* ranging from 0.94 to 0.99.

Studies related to the validity of the scale were conducted comparing individuals with borderline and neurotic disorders (Lerner and Lerner, 1980), patients with borderline personality disorder and schizophrenia (Lerner et al., 1981), people with borderline and narcissistic personality disorders (Farris, 1988), and patients with restrictive anorexia and bulimia (Brouilette, 1987; Piran and Lerner, 1987; Van-Der Keshet, 1988, all quoted by Lerner, 2005).

On the whole, findings confirmed that individuals with borderline disorders have specific primitive defensive levels both with respect to patients with neurotic disorders and patients with schizophrenia.

High scores on the LDS were also found in nailbiters in an Indian study (Arora et al., 2010) and in the parents of individuals with cocaine dependence in a Brazilian study (Pinheiro et al., 2001). To our knowledge, to date, no validation study has been carried out in European countries, and cross-cultural studies are not available.

Despite the promising findings, acknowledged also by Meyer et al. (2011) who indicated the LDS as a mature area for research, and the results from a recent clinical survey (Meyer et al., 2013) that rated all the LDS subscales accurate but Projective Identification, LDS is rarely used in clinical practice (Meyer et al., 2013). In addition, to date, no reference norms for non-clinical populations are available for all the defenses because only one published study (Baity et al., 2009) provided reference norms limited to Splitting, Devaluation, and Idealization subscales.

The current study aims to offer a contribution to this field by providing reference norms for all the LDS subscales and by investigating (a) the inter-rater reliability; (b) the most frequent coding mistakes in order to possibly improve the LDS coding guidelines; (c) the ability of the LDS to distinguish between individuals with neurotic and borderline levels of personality organization evaluated according to PDM-2 criteria, and a non-clinical group in an Italian sample; and (d) the association between Idealization and Devaluation with Reflection (Exner, 2003) and Space-fusion Rorschach responses (Rosso et al., 2015b, 2019; Rosso and Camoirano, 2019), which are the Rorschach structural variables assumed to be related to narcissistic personality traits. The main novelty of the present study consists in the fact that participants were evaluated by clinicians trained in psychoanalytic psychotherapy on the basis of PDM-2 dimensional diagnostic criteria, and not based on DSM categorical diagnostic criteria as in previous studies. In addition, to our knowledge, this is the first study exploring the association of the LDS with Reflection and Space-fusion responses.

It was expected that findings from this study would replicate substantial inter-rater reliability, while this study was exploratory regarding the analysis of the more frequent coding mistakes in an effort to possibly improve coding guidelines.

It was hypothesized that the LDS is able to discriminate borderline from a neurotic level of personality organization. Significant positive associations between Idealization and Reflection responses and between Devaluation and Space-fusion responses are hypothesized because Reflection responses are assumed to be markers of narcissistic traits also in healthy subjects (Exner, 2003), whereas Space-fusion responses were observed in more disordered narcissistically vulnerable individuals (Rosso et al., 2019).

## Materials and Methods

### Participants

Eighty clinical subjects and 80 non-clinical subjects participated in the study. Clinical subjects were self-referred outpatients who had undergone psychological assessment at a private clinical psychology service in northern Italy between 2017 and 2019. There were 37 females and 43 males ranging in age from 18 to 65 years (M age = 39.43 years ± 9.06) and in education from 8 to 23 years (M education = 13.90 years ± 4.05). Each of them had received a diagnosis according to the PDM-2: 40 subjects were outpatients with personality organized at a neurotic level, and 40 were outpatients with borderline personality organization. Each clinical subject, following intake interviews and psychological assessment, had been rated based on the Psychodiagnostic Chart-2 (PDC-2; Gordon and Bornstein, 2015, 2018) on P Axis and M Axis. Subjects in the Neurotic Level of Personality Organization group received scores ranging from 6 to 8 in the P Axis (M = 6.48 ±0.64) and scores ranging from 46 to 54 in the M Axis (M = 49.05 ± 2.56). Subjects in the Borderline Level of Personality Organization group received scores ranging from 3 to 5 in the P Axis (M = 4.23 ±0.66) and scores ranging from 31 to 46 in the M Axis (M = 38.25 ± 4.59).

Among the non-clinical subjects, 45 were women and 35 were men aged from 20 to 70 years (M age = 36.19 years ± 12.72) with a level of education ranging from 5 to 23 years (M education = 14.56 years ± 3.22).

They were drawn from an archival dataset of non-clinical individuals who had previously participated on a voluntary basis in a Rorschach normative study (Rosso et al., 2015a), reporting not having had any psychological, psychiatric, or neurological treatment, and not having used psychotropic medication or abused alcohol or illegal drugs. None of them obtained scores in the clinical range either on Beck Depression Inventory II (*M* = 3.69±2.41) or on Symptom Checklist-90-Revised (*M* = 43.74 ± 3.63).

No significant differences were found between clinical and non-clinical groups regarding sex (χ*2* = 1.60; *p* = 0.45), age (*t* = −1.854; *p* = 0.66), and education (*t* = 1.145; *p* = 0.254).

### Measures

The LDS (Lerner and Lerner, 1980) was applied to the Rorschach protocols, which were administered according to the Comprehensive System (CS, Exner, 2003).

For the purpose of this study, Reflection and human content responses were coded according to the CS, Space-fusion responses were coded according to Rosso and colleagues' criteria (Rosso et al., 2015b, 2019; Rosso and Camoirano, 2019), *Dr* (a Rorschach location score used when the area interpreted is small, seldom used and arbitrarily delimited) and *F(c)* (a Rorschach determinant used when the subject distinguishes forms within a shading area without using shading or uses the tones of shading within a colored area) were coded according to Rapaport, 1946.

When applying the LDS, all the responses containing a human figure must be evaluated. The human figure can be real or imaginary, whole, or with missing parts. Human detail contents must also be taken into account to rate the defense of Projective Identification (Lerner and Lerner, 1980, p. 259). Each human response can receive more than one LDS score.

Splitting involves the tendency to polarize descriptions of human content as indicated by the following markers: (1) two human content responses given in sequence are described with opposite affective tonalities; (2) a single human figure is described as divided into parts and reported as each part being the opposite of the other; (3) two human figures described in opposite ways are reported in the same response; (4) an implicitly idealized figure is diminished by negative features, or an implicitly devalued figure is embellished by other qualities.

Devaluation is ranked on a 5-point scale according to three dimensions: the degree to which the reality of the human figure is maintained, the space-time distancing, and the severity of the disparaging attribution. At level 1, the human figure is described in negative but socially acceptable terms, it is real, and it is not distant in space or time; at level 2, the figure is described in socially unacceptable negative terms, it is real even if it may be devoid of some of its parts, and it can be distant in time and space; at level 3, the figure is real but the response contains a distortion of the human form, it can be spaced out in time or space, and if it is negatively described, it is in socially acceptable terms; at level 4, the human dimension is still maintained but the human form is distorted, can be pushed away in time and space, and is described in negative and socially unacceptable terms; the difference with level 3 is the greater negativity of the description. At level 5, the human dimension is lost, the distorted form can be pushed away in time or space, and the figure can be described in neutral or negative terms.

Idealization is also rated on a 5-point scale along the same three dimensions. At level 1, the human dimension is maintained, the figure is not spaced out in time and space and is described in positive but not overly flattering terms; at level 2, there may be a time lag, and the figure is described with excessively positive tones; at level 3, the human figure can be described positively although not excessively and can be removed in space and/or time. Level 4 differs from level 3 because of the description in excessively positive terms, while at level 5 the human dimension is not maintained and the figure can be described in either neutral or positive terms and distanced in time and space.

Projective Identification is rated in confabulatory responses of inadequate formal level characterized by descriptions that neglect the real features of the stimuli and replace them with arbitrary fantasies and affects with an aggressive or sexual quality, or in human content responses (including Hd) with Dr localization, F(c) determinant, and aggressive connotation (acted or suffered).

Depending on the degree of distortion of reality, Denial is ranked along with three levels: high, medium, and low. High-level denial is shown in responses of adequate form quality through the disavowal of the impulse, or the intellectualization, or the minimization, or refutation of one's own response.

Medium-level denial is evident in responses of an adequate form quality that, however, present a logical, emotional contradiction, or an incongruous association that violates the reality principle. Low-level denial shows impairment in reality testing in two possible peculiar ways: an acceptable response is made inadequate because of the addition of an inappropriate percept or when the respondent fails to contemplate a facet of the blot that is obvious. Responses that include a bizarre incongruous combination also fall into this category.

Table 1 provides some examples for each defense and each level. Since idealization, devaluation, and denial are ranked on a continuum, in the present study these three variables were weighted according to rank, then collapsed into an overall derived weighted score for that category, as suggested by Hilsenroth et al. (1993, 1997).

**Table 1:** Examples of Rorschach responses coded according to Lerner Defense Scale *(LDS)* guidelines.

**Defense**	**Examples**
**Splitting**	“Two happy dancers rehearsing their favorite dance step” [W] followed by “Two angry men glaring at each other” [W] on Card II
	“A strange character, at the top he looks like an ugly monster with strange limbs ready to skewer, in the lower part he looks like a fluffy little chubby guy” [D3] on Card IX
	“Two women who look at each other, the one on the right has a sweet look and would like to shake hands with the one on the left who instead looks at her with hatred and would like to hurt her” [D9] on Card III
	“A nice dwarf” [D3] on Card IX
**Devaluation**	
Level 1	“A woman in an ugly dress” [D4] on Card I
Level 2	“A dirty woman who has rolled in the mud” [D4] on Card I
Level 3	“An ugly witch” [D4] on Card I
Level 4	“A scorched witch” [D4] on Card I
Level 5	“A mannequin without a head” [D4] on Card I
**Idealization**	
Level 1	“An elegant woman” [D4] on Card I
Level 2	“A very elegant woman with a beautiful evening dress” [D4] on Card I
Level 3	“An angel” [W] on Card I
Level 4	“A beautiful angel” [W] on Card I
Level 5	“An ancient statue of Venus”
**Projective Identification**	“The angry and hungry snowman who is about to come on me” [W] on Card IV “A man's profile who is going to hit someone” [Dd99: internal Dr in a small shading area] on Card IV
**Denial**	
High-level	“Two pacifists” [W] on Card II “Two Homo sapiens” [W] on Card II “Two angry Donald Ducks” [D9] on Card III “No, I do not remember I said that”
Medium-level	“A walking sleeping woman” [D9] on Card III
Low-level	“Two men raising a heart” [D1] on Card III

### Procedure

The first author selected Rorschach protocols from non-clinical and clinical archival datasets. Non-clinical protocols had been previously collected by graduate students after attending two academic courses on Rorschach testing (see Rosso et al., 2015a), while clinical protocols were administered by licensed clinical psychologists trained in Rorschach testing. Each subject gave written informed consent prior to administration, accepting that the Rorschach protocols would be used for research purposes, after being anonymized. The first author checked all the protocols to verify that they had been properly administered and coded, then, she anonymized and assigned them to the second and the third author, blinded to the protocol group the individual belonged to, so that the LDS could be applied. Altogether, 597 Rorschach responses were coded according to the LDS. In case of disagreement between the second and the third author, the coding decision was made by the first author.

## Results

Inter-rater reliability was calculated on all 160 Rorschach protocols. Altogether, 597 Rorschach responses were taken into account for coding, and 444 defenses were coded. On the whole, LDS percentage of agreement was 84%; it was, respectively, 100, 85, 81, 55, and 81% regarding Splitting, Devaluation, Idealization, Projective Identification, and Denial. Inter-rater reliability was substantial (Cohen's *k* = 0.79) for the five main defenses. Analysis of disagreement showed that 76% were due to errors of omission or commission, and in the remaining 24%, the errors were due to confusion between two different defenses. The former errors were mostly related to Devaluation at level 1 and high-level Denial, whereas all the errors of confusion concerned Projective Identification.

Preliminary analyses of the data indicated that the study variables were not normally distributed with skewness and kurtosis values falling outside the accepted range of ± 2 (George and Mallery, 2010), thus appropriate for non-parametric statistical tests. A Mann–Whitney *U*-test, performed to analyze the effect of sex on the LDS, did not find any significant effect (*ps* ranging from 0.109 to 0.957). Spearman's correlation analysis did not find any significant association between age and LDS scores (*ps* ranging from 0.272 to 0.946).

Comparisons between non-clinical and clinical groups, performed using the Kruskal–Wallis test, yielded significant differences regarding the total number of primitive defenses identified by the LDS (χ2 = 9.927; *p* = 0.007), Devaluation (χ2 = 8.067; *p* = 0.018), Projective Identification (χ2 = 10.543; *p* = 0.005), and Denial (χ2 = 11.982; *p* = 0.003). Then, pairwise group comparisons, using the Mann–Whitney U-test, were performed. The borderline group reported significantly higher scores than the non-clinical and neurotic groups on all four variables with effect sizes ranging from Cohen's *d* = 0.46 to Cohen's *d* = 0.73. Only the Denial scale discriminated between the non-clinical and neurotic group, with the latter reporting higher scores (*z* = −2.223; *p* = 0.026; *d* = 0.27). Regarding the other two Rorschach variables (Reflection and Space-fusion responses), subjects in the borderline group gave significantly more Space-fusion responses than the other two groups. Descriptive statistics, comparisons, and effect sizes are reported in Table 2.

**Table 2:** Descriptive statistics and comparisons between non-clinical and clinical groups.

	**NC** ***N*** **=** **80**		**NL** ***N*** **=** **40**		**BL** ***N*** **=** **40**			**Comparisons**		
**Variable**	**M**	**SD**	**M**	**SD**	**M**	**SD**		***Z***	***p***	***d***
P Axis			6.48	0.64	4.23	0.66	NL>BL	−7.918	<0.0001	3.46
M Axis			49.05	2.56	38.25	4.59	NL>BL	−7.714	<0.0001	3.02
R-LDS	3.45	2.37	4.18	3.19	3.85	2.79			n.s.	
Defenses	2.23	2.24	2.63	2.39	4.03	3.17	BL>NC BL>NL	−3.122 −1.990	0.002 0.047	0.67 0.50
S	0.03	0.16	0.08	0.27	0.10	0.38			n.s.	
WDV	4.03	4.34	4.68	4.96	7.23	6.04	BL>NC BL>NL	−2.766 −2.018	0.006 0.044	0.62 0.46
WI	1.86	3.01	1.78	3.41	1.93	3.92			n.s.	
PI	0.11	0.32	0.10	0.38	0.50	0.91	BL>NC BL>NL	−2.719 −2.590	0.007 0.010	0.63 0.62
WDN	0.46	1.12	0.85	1.76	1.85	2.69	NL>NC BL>NC	−2.279 −3.333	0.023 0.001	0.27 0.73
Reflection	0.34	0.76	0.50	0.82	0.28	0.60			n.s.	
S-fus	0.66	1.09	0.70	0.91	1.23	1.23	BL>NC BL>NL	−2.991 −2.038	0.003 0.042	0.49 0.50

*NC, non-clinical group; NL, Neurotic Level Personality Organization group; BL, Borderline Level Personality Organization group; M, mean; SD, standard deviation; Z, z statistic; p, p-value; d, Cohen's measure of effect size (|d| <0.20: negligible; |0.20| < d < |0.50|: small; |0.50| < d < |0.80| moderate; d > |0.80|: large); P Axis, Level of Personality Organization score on Psychodiagnostic Chart-2; M Axis, Mental Functioning score on Psychodiagnostic Chart-2; R-LDS = total responses coded according to the Lerner Defense Scale; Defenses: total defenses coded on the Lerner Defense Scale; WDV, Weighted Devaluation; WI, Weighted Idealization; PI = Projective Identification; WDN, Weighted denial; S-Fus, Space Fusion responses*.

A further comparison on the Denial subscales between groups showed that the neurotic group gave a higher number of high-level responses than subjects in the non-clinical group (*z* = −3.051; *p* = 0.002), while the borderline group gave a higher number of low-level responses compared with the neurotic group (*z* = −2.756; *p* = 0.006).

Since a significant correlation was found between the responses eligible for coding on the LDS and Weighted Devaluation (*rho* = 0.571; *p* < 0.0001), Weighted Idealization (*rho* = 0.453; *p* < 0.0001), Projective Identification (*rho* = 0.394; *p* < 0.0001), and Weighted Denial (*rho* = 0.394; *p* < 0.0001), comparisons were performed again after converting the score for each defense to a percentage score using the total number of responses eligible for coding on LDS as the denominator. Nine subjects (three in the non-clinical group, two in the Neurotic, and four in the Borderline-Level Personality Organization groups) were removed because they did not give any human response, so in these cases, the percentage could not be calculated. Results showed that subjects in the borderline-level group had a higher percentage of defenses coded on the LDS compared both with non-clinical subjects (*z* = −4.620; *p* < 0.0001) and with subjects in the neurotic level group (*z* = −4.103; *p* < 0.0001) with large effect sizes (Cohen's *d*, respectively, 1.02 and 1.04). Effect sizes were in the moderate range (Cohen's *d* ranging from 0.54 to 0.69) regarding the defenses of Devaluation, Projective Identification, and Denial, with subjects in the borderline-level group reporting higher scores than the other two groups. Results are reported in Table 3.

**Table 3:** Comparisons between non-clinical and clinical groups on LDS variables (after converting the score for each defense to a percentage using the total number of responses eligible for coding on LDS as the denominator).

	**NC** ***N*** **=** **77**		**NL** ***N*** **=** **38**		**BL** ***N*** **=** **36**			**Comparisons**		
**Variable**	**M**	**SD**	**M**	**SD**	**M**	**SD**		***z***	***p***	***d***
Defenses%	0.61	0.43	0.63	0.38	1.04	0.41	BL>NC BL>NL	−4.620 −4.103	<0.0001 <0.0001	1.02 1.04
WDV%	1.14	1.19	1.31	1.35	2.09	1.58	BL>NC BL>NL	−3.030 −2.185	0.002 0.029	0.69 0.54
WI%	0.50	0.79	0.35	0.60	0.56	1.01			n.s.	
PI%	0.024	0.072	0.017	0.063	0.11	0.22	BL>NC BL>NL	−2.825 −2.757	0.005 0.006	0.59 0.66
WDN%	0.14	0.43	0.17	0.28	0.46	0.68	NL>NC BL>NC	−2.223 −3.395	0.026 0.001	0.58 0.60

*NC, non-clinical group; NL, Neurotic-Level Personality Organization group; BL, Borderline-Level Personality Organization group; M, mean; SD, standard deviation; Z, z statistic; p, p-value; d, Cohen's measure of effect size (|d| <0.20: negligible; |0.20| < d < |0.50|: small; |0.50| < d < |0.80| moderate; d > |0.80|: large); Defenses%, percentage of defenses coded on the total responses eligible for coding on the LDS; WDV%, percentage of Weighted Devaluation on the total responses eligible for coding on the LDS; WI%, percentage of Weighted Idealization on the total responses eligible for coding on the LDS; PI%, percentage of Projective Identification on the total responses eligible for coding on the LDS; WDN%, percentage of Weighted denial on the total responses eligible for coding on the LDS*.

Partial correlation analysis, controlling for the number of responses coded on the LDS, was performed to investigate the association between LDS scores, PDC-2 scores on P Axis and M Axis, and Reflection and Space-Fusion Rorschach responses. Devaluation and Projective Identification were correlated with P Axis (respectively, *rho* = −0.336 and *rho* = −0.290), and with M Axis (respectively, *rho* = −0.376 and *rho* = −0.282). Defenses did not correlate with each other, but Denial correlated with Projective Identification (*rho* = −0.292). Idealization correlated with Reflection responses (*rho* = 0.232), whereas no defense correlated with Space-fusion responses, although the latter correlated negatively both with P Axis (*rho* = −0.327) and with M Axis (*rho* = −0.255). Results are shown in Table 4.

**Table 4:** Partial correlations (controlling for coded responses on the Lerner Defense Scale) among Psychodiagnostic Chart-2 (PDC-2), LDS, and Rorschach variables.

	**P Axis**	**M Axis**	**S**	**WDV**	**WI**	**PI**	**WDN**	**Reflection**
P Axis	–							
M Axis	0.928^***^	–						
S	−0.077	−0.098	–					
WDV	−0.336^**^	−0.376^**^	−0.003	–				
WI	−0.059	−0.013	0.131	0.092	–			
PI	−0.290^**^	−0.282^*^	−0.027	−0.068	−0.143	–		
WDN	−0.193	−0.186	0.122	0.064	−0.087	0.292^**^	–	
Reflection	0.172	0.181	0.121	0.025	0.232^*^	−0.077	−0.140	–
S-fus	−0.327^**^	−0.255^*^	−0.068	0.132	−0.073	0.184	−0.029	0.055

*P Axis, Level of Personality Organization score on Psychodiagnostic Chart-2; M Axis, Mental Functioning score on Psychodiagnostic Chart-2; S, Splitting; WDV, Weighted Devaluation; WI, Weighted Idealization; PI, Projective Identification; WDN, Weighted denial; S-Fus, Space Fusion responses*.

A further partial correlation between S-fusion responses and Devaluation subscales showed that S-fusion responses correlated significantly with Devaluation at level 1 (*rho* = 0.462; *p* < 0.0001), level 2 (*rho* = 0.379; *p* = 0.001), and level 4 (*rho* = 0.280; *p* = 0.017).

## Discussion

Findings confirmed a more than satisfactory level of inter-rater agreement although some issues emerged with regard to the scoring of Projective Identification. An analysis of the coding errors revealed that the most crucial issue was confusion between Devaluation and Projective Identification when the associative elaboration involved material with aggressive meaning. Errors were mostly due to the fact that the rater had not correctly understood the particular confabulatory quality of the Projective Identification response. Another critical dilemma was whether or not to code Projective Identification when a human detail without aggressive content, such as “eyes,” is interpreted in a Dr location with an F(c) determinant. In the current study, according to LDS scoring guidelines, we did not score this kind of response; however, it might be interesting for further studies to investigate whether or not validity improves when coding the “eyes responses.” Concerning Idealization and Devaluation, omission and commission errors were due to the fact that responses such as “astronaut” or “two women dancing together” or “waiters” are indicated in some studies (e.g., Lerner and Van-Der Keshet, 1995; Lerner, 2005) as signs of Idealization or Devaluation without a very clear rationale, so that sometimes rating the highest levels of Idealization and Devaluation is challenging. To overcome these doubts, a scoring system providing more examples could be useful.

It was hypothesized that the LDS is able to discriminate individuals in the neurotic personality organization group from persons in the borderline level of personality organization group. Results showed that some subscales, namely, Devaluation, Projective Identification, and Denial, were able to discriminate between the two groups, while Idealization and Splitting were not. In particular, Devaluation and Projective Identification correlated significantly and negatively both with P Axis and M Axis, supporting the validity of the LDS in distinguishing between developmental levels of personality organization.

Devaluation is a frequently used defense mechanism by individuals with a borderline level of personality organization and a fragile sense of self. It protects them against having to recognize the need for the Other, thus defending themselves against feelings of envy and fear of abandonment. The positive correlation found between Devaluation and Space-fusion Rorschach responses assumed to be a sign of marked narcissistic vulnerability in personality disordered individuals (Rosso and Camoirano, 2019), offers further support to the hypothesis that Devaluation is a marker of malignant narcissism (Kernberg, 2004).

Projective identification is a primitive defense mechanism typically used by individuals with a borderline level of personality organization: they project intolerable intrapsychic experiences onto another person, often a close individual, feeling empathy with what they project, trying to control the other in a continuing effort to defend themselves against the intolerable experience, and, unconsciously, in actual interaction with the other, leading the individual to experience what has been projected onto him/her (Kernberg, 1987). Not surprisingly, in the current study, Projective Identification correlated with the most primitive levels of Denial.

With regard to the Denial subscale, results showed that subjects with neurotic-level personality organization used high-level Denial more frequently compared to the non-clinical group, while individuals with borderline-level personality organization more frequently made use of low-level Denial than neurotic and non-clinical subjects did. This finding, which is in line with previous studies (see Lerner, 2005 for a review), raises some doubts about whether it is appropriate to include neurotic forms of negation, intellectualization, or minimization in a scale designed to rate primitive defenses, especially if a weighted score is used because it could be misleading, above all, in the protocols that have a high number of responses rated as a high-level denial. For example, in our analyses, when the weighted score was used, Denial did not correlate with either P Axis or M Axis, while the low-level Denial subscale correlated significantly and negatively with P Axis (*rho* = −0.236; *p* = 0.036). In the current study, LDS subscales did not correlate with each other, except for a significant correlation found between Projective Identification and Denial. A further correlation analysis showed that particularly medium-level and low-level Denial correlated with PI (*rho* = 0.285; *p* = 0.011 and *rho* = 0.257; *p* = 0.022 respectively), while no correlation emerged between high-level Denial and Projective Identification (*rho* = 0.099; *p* = 0.385). This finding, which needs replication studies, seems to suggest that low-level Denial and Projective Identification correlated with each other in that they imply a more impaired mental functioning associated with a more severe reality distortion due to the eruption from the primary process that disrupts the ego functions of secondary process thinking.

Our findings regarding Idealization support the hypotheses put forth in a previous study (Lerner and Van-Der Keshet, 1995). According to Kernberg's (1980) assumption, idealization falls on a continuum from pathological to normal, and it implies also non-defensive aspects, including a precondition for feelings of mature love. Results from the current study further support the hypothesis according to which the Idealization subscale is more sensitive to the adaptive aspects of an idealization than to the defensive ones. The positive correlation between Reflection responses and Idealization offers further support to this hypothesis, being Reflection responses are also an indicator of adaptive narcissism (Exner, 2003).

Contrary to some previous findings (Lerner and Lerner, 1980; Lerner et al., 1981; Farris, 1988), in the current study Splitting did not distinguish between non-clinical and clinical groups. Only 2.5% of the non-clinical subjects and 7.5% of both the neurotic and the borderline groups gave a splitting response. It might be assumed that this result depends on the fact that in the borderline group, only five out of 40 subjects (12.5%) were rated at the lowest borderline level on the P Axis. Based on this supposition, results might confirm that splitting is a defense mostly used by more severely disturbed individuals with personality organized at the lowest borderline personality level.

Finally, this study offers the first reference norms for a non-clinical population for all five main defenses (see Table 2). A previous study (Baity et al., 2009) offered norms for three out of the five defenses, namely, Splitting, Devaluation, and Idealization. A comparison between our results and Baity et al.'s findings did not show significant differences (Cohen's *d* respectively −0.38 for Splitting, −0.25 for Devaluation, and −0.01 for Idealization).

In conclusion, the current study provides suggestions for improving the scoring system and offers further support to the validity of the LDS. Specifically, Devaluation, Projective Identification, and low-level Denial subscales were able to discriminate between neurotic and borderline levels of personality organization. In addition, the current study provides reference norms available for non-clinical populations, which could encourage broader use of the LDS in clinical practice as well as in research, including psychotherapy outcome studies.

## Data Availability Statement

The raw data supporting the conclusions of this article will be made available by the authors, without undue reservation.

## Ethics Statement

Ethical review and approval was not required for the study on human participants in accordance with the local legislation and institutional requirements. The patients/participants provided their written informed consent to participate in this study.

## Author Contributions

AR designed the study, coordinated data collection, performed the statistical analyses, and prepared the first draft of the article. CA and AC contributed to the search for references, coded the Rorschach protocols, cooperated in performing the statistical analyses, and contributed to the final version. All authors contributed to the article and approved the submitted version.

## Conflict of Interest

The authors declare that the research was conducted in the absence of any commercial or financial relationships that could be construed as a potential conflict of interest.

## Publisher's Note

All claims expressed in this article are solely those of the authors and do not necessarily represent those of their affiliated organizations, or those of the publisher, the editors and the reviewers. Any product that may be evaluated in this article, or claim that may be made by its manufacturer, is not guaranteed or endorsed by the publisher.
